# MicroRNAs in Tumor Endothelial Cells: Regulation, Function and Therapeutic Applications

**DOI:** 10.3390/cells12131692

**Published:** 2023-06-22

**Authors:** Yuan Gu, Maximilian A. Becker, Luisa Müller, Katharina Reuss, Frederik Umlauf, Tianci Tang, Michael D. Menger, Matthias W. Laschke

**Affiliations:** Institute for Clinical & Experimental Surgery, Saarland University, 66421 Saar, Germany; maximilian_arno.becker@uni-saarland.de (M.A.B.); s8llmu12@stud.uni-saarland.de (L.M.); s8kareus@stud.uni-saarland.de (K.R.); s8frumla@stud.uni-saarland.de (F.U.); tianci.tang@uks.eu (T.T.); michael.menger@uks.eu (M.D.M.); matthias.laschke@uks.eu (M.W.L.)

**Keywords:** angiogenesis, cancer, miRNA, tumor endothelial cells, tumor microenvironment, therapy

## Abstract

Tumor endothelial cells (TECs) are key stromal components of the tumor microenvironment, and are essential for tumor angiogenesis, growth and metastasis. Accumulating evidence has shown that small single-stranded non-coding microRNAs (miRNAs) act as powerful endogenous regulators of TEC function and blood vessel formation. This systematic review provides an up-to-date overview of these endothelial miRNAs. Their expression is mainly regulated by hypoxia, pro-angiogenic factors, gap junctions and extracellular vesicles, as well as long non-coding RNAs and circular RNAs. In preclinical studies, they have been shown to modulate diverse fundamental angiogenesis-related signaling pathways and proteins, including the vascular endothelial growth factor (VEGF)/VEGF receptor (VEGFR) pathway; the rat sarcoma virus (Ras)/rapidly accelerated fibrosarcoma (Raf)/mitogen-activated protein kinase kinase (MEK)/extracellular signal-regulated kinase (ERK) pathway; the phosphoinositide 3-kinase (PI3K)/AKT pathway; and the transforming growth factor (TGF)-β/TGF-β receptor (TGFBR) pathway, as well as krüppel-like factors (KLFs), suppressor of cytokine signaling (SOCS) and metalloproteinases (MMPs). Accordingly, endothelial miRNAs represent promising targets for future anti-angiogenic cancer therapy. To achieve this, it will be necessary to further unravel the regulatory and functional networks of endothelial miRNAs and to develop safe and efficient TEC-specific miRNA delivery technologies.

## 1. Introduction

Cancer is a leading cause of global death [1]. Although the diagnosis and therapy of some tumor types have been considerably improved in recent years, novel efficient treatment options are still urgently needed. Such a promising option is the inhibition of angiogenesis, which may be performed as monotherapy or in combination with other therapeutic approaches [2,3].

**Figure 1:**
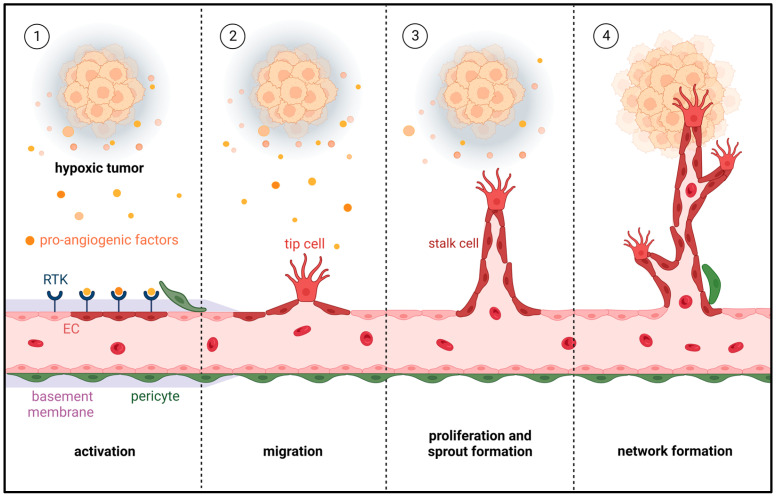
Process of tumor angiogenesis. Once a tumor grows beyond a few cubic millimeters, hypoxia induces the release of pro-angiogenic factors from tumor cells into the surrounding microenvironment (①). The binding of these factors to receptor tyrosine kinases (RTKs) activates ECs to secrete proteases that cause basement membrane degradation and pericyte detachment (①). The activated EC tip cells then migrate towards the tumor (②), while trailing EC stalk cells proliferate to form vascular sprouts (③). The sprouts develop branches and finally interconnect with each other into new microvascular networks, which support further tumor growth (④).

Angiogenesis is defined as the growth of new blood vessels from pre-existing ones and is well known as one of the major cancer hallmarks, as defined by Hanahan and Weinberg [4]. It typically occurs when a tumor reaches 1–2 mm^3^ in volume and can no longer be adequately supplied with oxygen and nutrients via diffusion [5]. During angiogenesis, endothelial cells (ECs) lining the lumen of blood vessels are activated by pro-angiogenic factors that are released from hypoxic tumor cells, as well as other components of the tumor microenvironment (TME) [6]. These pro-angiogenic factors include vascular endothelial growth factor (VEGF), basic fibroblast growth factor (bFGF), angiopoietins, transforming growth factor (TGF)-β and placental-derived growth factor (PDGF) [7,8]. Upon their binding to cell surface receptors, ECs are stimulated to proliferate, migrate, form vascular sprouts and, ultimately, assemble into new microvascular networks within the tumor tissue [6] (Figure 1). This process is essential for tumor survival, growth and metastasis.

Compared to blood vessels within normal tissues, tumor vessels are irregularly organized, fragile and leaky [5]. Hence, it is reasonable that tumor ECs (TECs) differ from normal ECs in terms of morphology and function. TECs are highly heterogeneous and sensitive to certain growth factors, such as VEGF, but resistant to serum starvation and anti-cancer drugs, including 5-fluorouracil and paclitaxel [9]. Moreover, they are characterized by impaired endothelial barrier function, as well as increased angiogenic and metabolic activities [10]. All these features are probably due to their genetic abnormality. In fact, previous studies reported that TECs exhibit markedly different expression patterns of genes and non-coding RNAs when compared to normal ECs [11,12,13,14].

**Figure 2:**
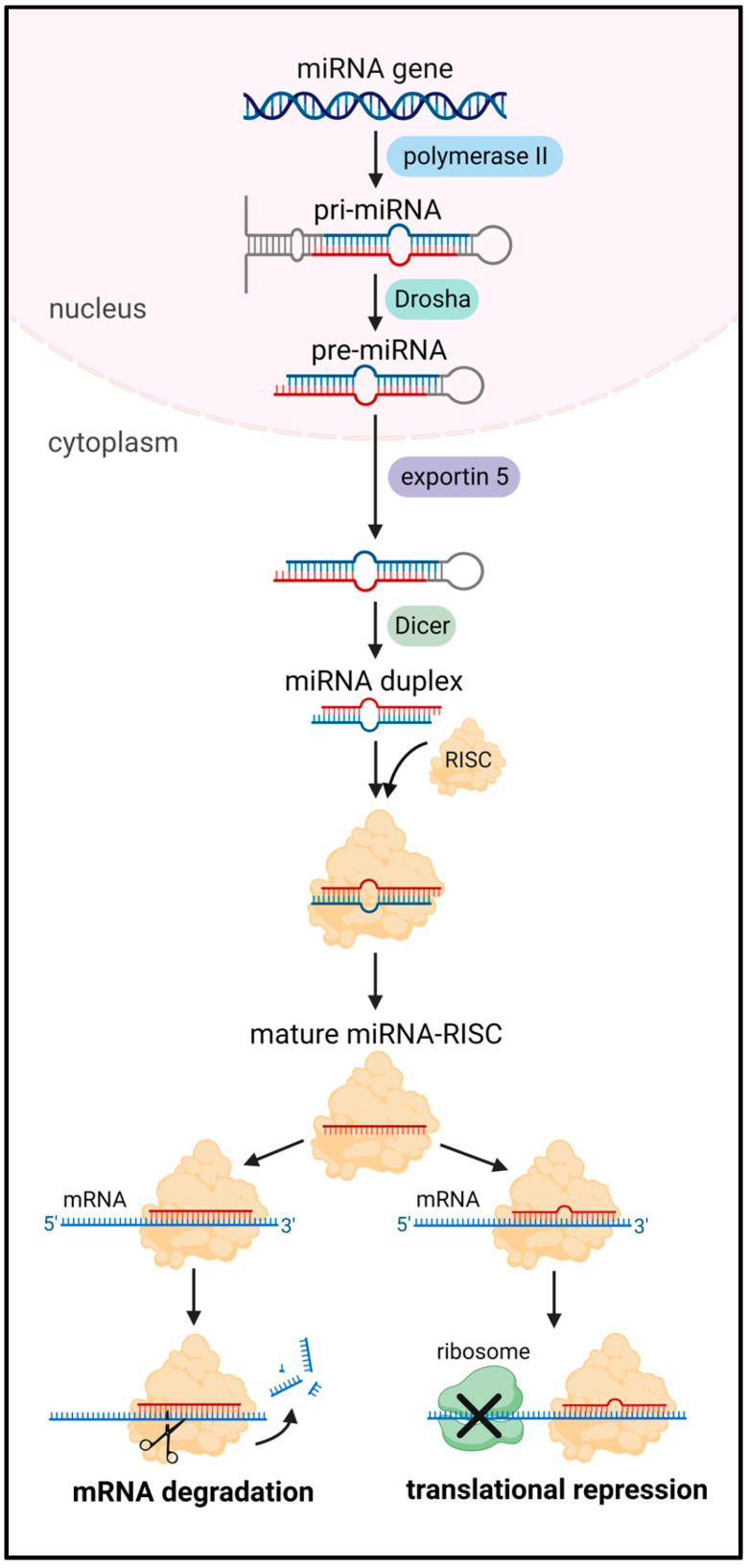
MiRNA biogenesis and function. An miRNA gene is generally transcribed to pri-miRNA by RNA polymerase II. Following the cleavage of pri-miRNA by Drosha, the resulting pre-miRNA is transported by exportin-5 out of the nucleus into the cytoplasm. Further cleavage by Dicer results in the generation of an miRNA duplex, which associates with the RISC. This association facilitates the discarding or degradation of one strand of the duplex. The remaining mature miRNA then binds completely or partially to its target transcript, leading to mRNA degradation or translation repression.

MicroRNAs (miRNAs), a specific type of non-coding RNA molecule of 18–24 nucleotides, have been shown to be implicated in the abnormality of TECs and the development of tumor vasculatures [15]. They are mainly transcribed by RNA polymerase II from miRNA genes, introns of protein-coding genes or polycistronic transcripts. A small subset of miRNAs can also be transcribed by RNA polymerase III [16,17]. The resulting long hairpin-like primary transcript (pri-miRNA) with thousands of nucleotides is further processed within the nucleus by RNase III Drosha into 70–90-nucleotide stem-loop precursor miRNA (pre-miRNA). The pre-miRNA is then exported by exportin 5 to the cytoplasm, where it is cleaved by RNase III Dicer into miRNA duplex. Finally, the miRNA duplex is loaded onto the RNA-induced silencing complex (RISC) and unwound into the single-stranded mature form and its complementary strand, which is normally degraded [18] (Figure 2). The nomenclature of the mature miRNA is determined by the directionality of the miRNA strand. The 5p strand (miR-5p) arises from the 5′ side of the pre-miRNA, while the 3p strand (miR-3p) originates from the 3′ side [19]. The mature miRNA in the RISC is able to guide the complex to its messenger RNA (mRNA) targets, usually by base pairing with their 3′untranslated regions (UTRs). This leads to the degradation or translation inhibition of target mRNAs depending on the degree of miRNA–mRNA complementarity [19] (Figure 2). Of note, non-canonical binding sites of miRNAs in mRNA regions have also been identified, including the 5′UTR, coding sequence and promoter regions [15,19]. Accordingly, each miRNA has the ability to target multiple genes and, thus, serves as a powerful regulator of diverse cellular processes, such as apoptosis, proliferation and migration [15]. Importantly, miRNAs play a pivotal role in maintaining physiological homeostasis, and their dysregulation has been strongly associated with a broad spectrum of human diseases, such as cancer [20].

Although it is known that miRNAs in different cell types of the TME are capable of modulating tumor angiogenesis [15], we exclusively focus in this systematic review on miRNAs in ECs (also called endothelial miRNAs) that are involved in the regulation of TEC angiogenic activity. In detail, we elucidate the different mechanisms regulating their expression, describe their functions and targets in tumor angiogenesis modulation, illustrate their potential therapeutic applications along with associated challenges and provide insights into future directions of the field.

## 2. Endothelial miRNAs Involved in Tumor Angiogenesis

**Figure 3:**
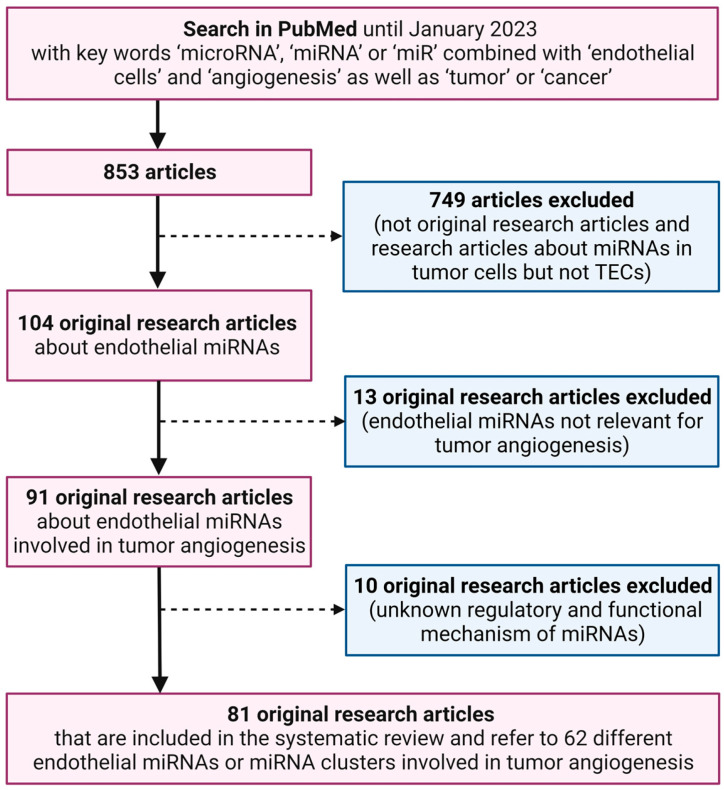
Flow diagram displaying the systematic literature search for the present review. This literature search was performed to identify original research articles focusing on endothelial miRNAs regulating tumor angiogenesis.

In order to retrieve all published papers that focus on endothelial miRNAs regulating tumor angiogenesis, a systematic literature search was performed in the PubMed database until January 2023, as shown in Figure 3. The key words for this search included ‘microRNA’, ‘miRNA’ or ‘miR’ combined with ‘endothelial cells’ and ‘angiogenesis’, as well as ‘tumor’ or ‘cancer’. Only original research articles written in English, focusing on miRNAs in ECs and investigating the effects of endothelial miRNAs on tumor angiogenesis were included. 

We detected 81 original research articles, which fulfilled the above-mentioned inclusion criteria. These articles referred to 62 different endothelial miRNAs or miRNA clusters that are involved in tumor angiogenesis. The names of these miRNAs and the mechanisms regulating their expression, as well as their functional targets and effects, are listed in Table 1.

### 2.1. Regulation of miRNA Expression in ECs

**Figure 4:**
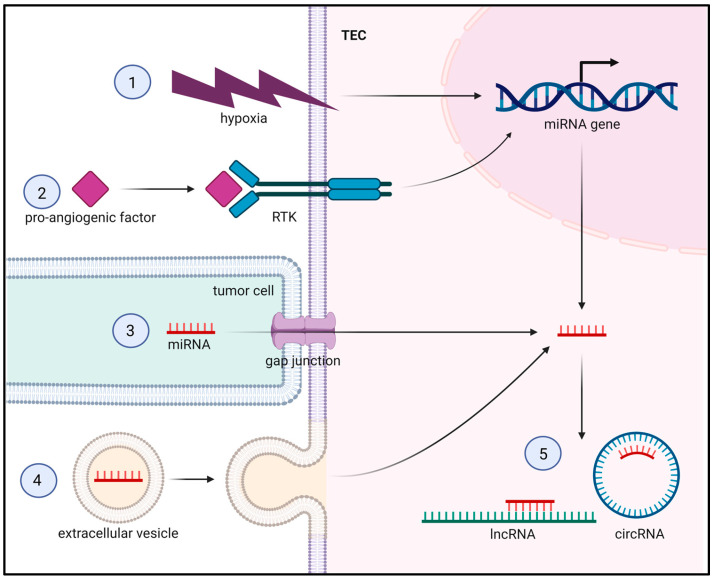
Regulation of miRNA expression in TECs. ① and ②: Hypoxia and pro-angiogenic factors in the TME regulate the expression of miRNAs in TECs. ③ and ④: MiRNAs are transferred into TECs from other cell types of the TME, such as tumor cells, via gap junctions or extracellular vesicles. ⑤: Intracellular miRNAs are sponged by lncRNAs and circRNAs.

Accumulating evidence suggests that the expression profile of miRNAs in TECs differs from that in normal ECs [13,14]. This dysregulation is triggered by the TME via multiple mechanisms, as outlined in the following subsections by means of selected, exemplary miRNAs, and summarized in Figure 4.

#### 2.1.1. Hypoxia

Hypoxia is a key microenvironmental feature of the majority of solid tumors [102]. It drives tumor angiogenesis, metastasis, immunosuppression and treatment resistance by regulating various cell types of the TME, including tumor cells, fibroblasts and ECs [102,103]. The hypoxic TME stimulates EC angiogenesis mainly via the activation of hypoxia-inducible factors (HIFs), which are highly conserved transcriptional factors regulating a multitude of genes and non-coding RNAs [104,105]. Recently, we could demonstrate in vitro that HIF1α activation in human dermal microvascular ECs (HDMECs) exposed to hypoxia inhibits the transcription of miR-186-5p (previous name: miR-186), which may explain the downregulation of this miRNA in TECs of human non-small-cell lung cancer (NSCLC) samples [49]. This finding is consistent with a recent study reporting that the expression level of miR-186 in human umbilical vein ECs (HUVECs) is decreased under hypoxic conditions [106]. The downregulation of miR-186 due to hypoxia, in turn, promoted the angiogenic activity of ECs by upregulating protein kinase C, a bona fide target of miR-186 [49]. 

#### 2.1.2. Pro-Angiogenic Factors

Pro-angiogenic factors in the TME stimulate angiogenesis mainly by binding to their receptors on ECs and activating intracellular downstream signaling pathways. However, they can also exert their effects by regulating the expression of miRNAs in ECs.

VEGF is one of the most potent pro-angiogenic growth factors. Among the VEGF family members, which include VEGF-A, VEGF-B, VEGF-C, VEGF-D, VEGF-E and placental growth factor (PlGF), VEGF-A (often abbreviated as VEGF) plays a dominant role in regulating angiogenesis and blood vessel permeability [107]. It has been reported that VEGF secreted by U87 glioblastoma cells downregulates the expression of miR-125b-5p (miR-125b) in human brain microvascular ECs (HBMECs), which consequently stimulates EC angiogenesis, because miR-125b acts as an angiogenesis inhibitor [27]. Moreover, the expression of anti-angiogenic miR-1-3p (miR-1) was downregulated in VEGF-stimulated ECs and ECs isolated from the lungs of VEGF transgenic mice [108], as well as ECs isolated from mouse NSCLC tumors [21]. EC-specific miR-1 overexpression mediated by lentivirus vectors or transgenic methods suppressed tumor growth and angiogenesis in several mouse models of NSCLC [21]. These findings suggest crucial clinical significance of miR-1 in the anti-angiogenic treatment of NSCLC. On the contrary, VEGF upregulated the expression of miR-296-5p (miR-296) in HBMECs in culture, which may explain the elevated level of this miRNA in TECs isolated from human gliomas. Furthermore, the inhibition of miR-296 with antagomirs reduced vascularization in tumor xenografts [74]. The upregulation of endothelial miR-296 by VEGF was later confirmed by Kim et al. in HUVECs [109].

Interleukin (IL)-1β, a well-known pro-inflammatory cytokine, serves as an important pro-angiogenic factor in the TME [110]. In ECs, it mediates the phosphorylation and degradation of the inhibitor of nuclear factor κB (IκB) kinase [111]. Subsequently, IκB-free nuclear factor κB (NF-κB) translocates into the nucleus, where it controls the transcription of mRNAs as well as miRNAs [112,113]. In a previous study, we found that IL-1β released by NSCLC cells activates NF-κB and, thus, suppresses the expression of miR-22-3p (miR-22) in HDMECs co-cultured with NSCLC cells. This mechanism possibly contributes to the observed downregulation of miR-22 in TECs of human NSCLC samples [59]. These findings are in line with previous studies showing that IL-1 downregulates the expression of miR-22 in primary cultured chondrocytes [114]. Moreover, NF-κB directly binds to the miR-22 promoter and inhibits the transcription of this miRNA in 182R-6 breast cancer cells [115]. In addition, the overexpression of miR-22 in ECs resulted in the inhibition of NSCLC angiogenesis and growth, suggesting that this miRNA holds promise as a therapeutic target for anti-angiogenic cancer treatment [59]. 

TGF-β is a prominent member of a large family consisting of 33 multifunctional cytokines, including TGF-β isoforms, activins and bone morphogenetic proteins [116]. It regulates a plethora of cellular processes, such as proliferation, motility and differentiation during organ development and homeostasis, while its dysregulation has been linked to multiple diseases, including fibrosis, vascular pathologies and cancer [116]. It is noteworthy that TGF-β has also been proposed to modulate angiogenesis. Its angiogenic and angiostatic effects on ECs are dose- and context-dependent in vitro [117]. However, it serves as a potent angiogenesis inducer in vivo [117]. McCann et al. [76] recently reported that TGF-β is capable of reducing the transcription of miR-30c-5p (miR-30c) in ECs. The vascular tropic nanoparticle-mediated delivery of miR-30c antagomirs promoted E0771 mammary tumor angiogenesis and growth, whereas miR-30c mimics showed the opposite effects in vivo. It is worth noting that the downregulation of miR-30c by TGF-β has also been observed in other cell types, including primary hepatic stellate cells [118], renal tubular epithelial cells [119], cardiac fibroblasts [120] and ovarian cancer cells [121].

#### 2.1.3. Gap Junctions

Gap junctions, which are composed of transmembrane connexin hexamers, represent membrane channels that mediate the direct transfer of small molecules, such as ions, amino acids, secondary messengers and metabolites, between adjacent cells in solid tissues [122]. They play pivotal roles in a wide range of both physiological and pathological processes [123]. In particular, it has been shown that gap junctions mediate the interaction between tumor cells and ECs and are therefore directly involved in the induction of tumor angiogenesis [124,125]. This view is further supported by a recent study showing that miR-5096 is transported from glioblastoma cells to ECs via gap junctions, leading to EC tube formation [86]. Of interest, gap junctions also play a role in the transfer of miR-5096 from glioblastoma cells to astrocytes. As a consequence, miR-5096 promotes glioma invasiveness, while the underlying mechanism needs further elucidation [126].

#### 2.1.4. Extracellular Vesicles (EVs)

EVs are lipid bilayer-encapsulated particles that are released by almost all types of cells [127]. They serve as vesicles for the exchange of proteins, lipids and nucleic acids between cells, which is considered as an important mechanism of intercellular communication [127]. Based on their size and origin, EVs are generally categorized into exosomes and microvesicles [128]. Exosomes originate from endosomes, and their size ranges from 50 to 150 nm. In contrast, microvesicles with a diameter of up to 1000 nm emerge from the plasma membrane [128]. Out of the 62 miRNAs or miRNA clusters listed in this review, 37 have been demonstrated to be transferred from epithelial cells, tumor cells or cancer stem cells into ECs through EVs, which subsequently modulate tumor angiogenesis. Examples of such miRNAs include miR-1229-3p (miR-1229) [24], miR-1246 [26], miR-21-5p (miR-21) [52], miR-221-3p (miR-221) [62], miR-25-3p (miR-25) [69], miR-9-5p (miR-9) [93] and miR-92a-3p (miR-92a) [95], which can be delivered from colorectal cancer cells to ECs via EVs and promote angiogenesis. It is important to note that the origin of miRNA-containing EVs is often not limited to a single type of cancer cell. For instance, microvesicles derived from NSCLC cells, melanoma cells, pancreatic cancer cells and glioblastoma cells have also been shown to upregulate endothelial miR-9 [93]. Moreover, the intratumoral injection of miR-9 antagomirs inhibited the vascularization and growth of the HM7 colorectal tumor and LLC lung carcinoma [93]. These findings suggest that EVs derived from the TME play a significant and major role in regulating endothelial miRNAs and tumor angiogenesis.

#### 2.1.5. Long Non-Coding RNAs (lncRNAs) and Circular RNAs (circRNAs)

Endothelial miRNAs can also be regulated by lncRNAs and circRNAs. lncRNAs are a class of single-stranded RNA that lack protein-coding capacity, with a length longer than 200 nucleotides [129], while circRNAs are single-stranded non-coding RNAs with a covalently closed-loop structure [130]. Both lncRNAs and circRNAs possess binding sites for miRNAs and serve as miRNA sponges. Sponged miRNAs are incapable of interacting with their target mRNAs. As a consequence, the target genes of miRNAs are positively regulated by lncRNAs and circRNAs [131,132]. It has been reported that endothelial miR-29a-3p (miR-29a) is sponged by lncRNA H19, which is upregulated in glioma microvessels and ECs cultured in glioma cell-conditioned medium. Accordingly, the knockdown of lncRNA H19 resulted in miR-29a upregulation and the downregulation of its target, vasohibin 2 (*VASH2*), in ECs, ultimately leading to the inhibition of glioma-induced EC angiogenesis in vitro [73]. The sequestration of miR-29a by lncRNA H19 has also been observed in many cancer cell types, such as breast cancer cells [133], clear cell renal cell carcinoma cells [134] and osteosarcoma cells [135]. In addition, endothelial miR-138-5p (miR-138) was targeted by circ_002136 and downregulated in ECs cultured in U87 glioblastoma cell-conditioned medium (GECs). It suppressed GEC angiogenesis by targeting *SOX13* and subsequentially increasing SPON2 transcription [33].

### 2.2. Function of Endothelial miRNAs in Tumor Angiogenesis

**Figure 5:**
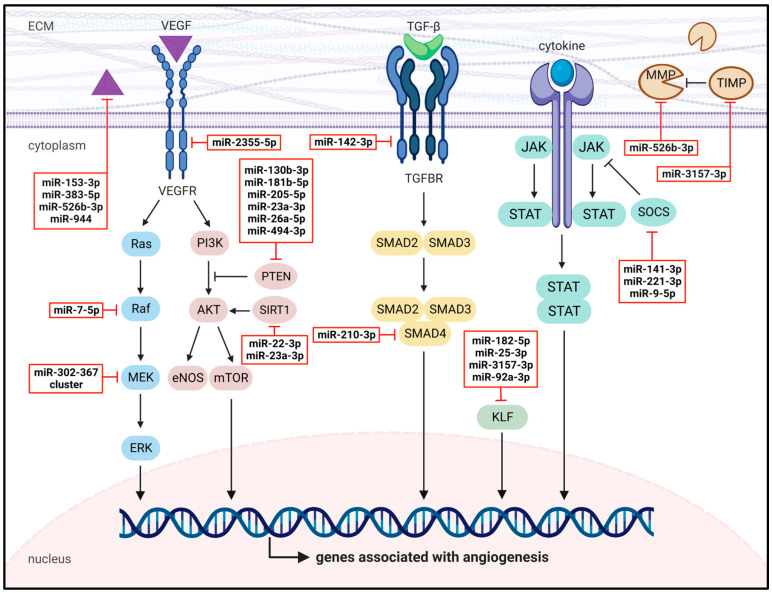
Function of endothelial miRNAs in tumor angiogenesis. Endothelial miRNAs regulate the aberrant angiogenic activity of TECs by targeting important angiogenesis-related signaling pathways and proteins, including the VEGF/VEGFR, Ras/Raf/MEK/ERK, PI3K/AKT and TGF-β/TGFBR pathways, as well as KLFs, SOCS and MMPs/TIMPs. Representative endothelial miRNAs are shown in red boxes.

Endothelial miRNAs are considered potent regulators of tumor angiogenesis due to their capacity to target multiple genes associated with angiogenesis. Indeed, they exert pro- or anti-angiogenic effects by regulating diverse angiogenesis-related signaling pathways and proteins, as outlined in the following subsections by means of selected, exemplary miRNAs, and summarized in Figure 5.

#### 2.2.1. VEGF/VEGF Receptor (VEGFR) Pathway

Among the three VEGFR family members, VEGFR2 plays a dominant role in regulating EC proliferation, migration and survival, as well as vascular permeability [136]. The VEGF/VEGFR pathway is essential for tumor angiogenesis and, thus, widely considered an important target for anti-angiogenic therapy. Many endothelial miRNAs exert their anti-angiogenic effects by targeting this pathway. For instance, miR-153-3p [43], miR-383-5p [81] and miR-526b-3p [87] have been reported to target *VEGF*, while miR-944 targets *VEGFC* in ECs [100]. Notably, the direct binding of miR-383-5p to VEGF mRNA has been confirmed by Han et al. in ECs [137] and demonstrated in other cell types, including bone-marrow-derived mesenchymal stem cells [138] and lung adenocarcinoma cells [139]. Moreover, miR-2355-5p (miR-2355) targets *VEGFR2* in ECs [68]. In another study, miR-2355 was found to target *VEGFR2* endothelial colony-forming cells isolated from the peripheral blood of patients with coronary artery disease [140]. Additionally, a larger number of endothelial miRNAs have been shown to regulate the VEGF/VEGFR pathway in an indirect manner, such as miR-125b-5p (miR-125b), miR-144-3p (miR-144) and miR-940, by targeting Myc-associated zinc finger protein (*MAZ*), F-box and WD repeat domain containing 7 (*FBXW7*), and v-ets erythroblastosis virus E26 oncogene homolog 1 (*ETS1*), respectively [27,39,99].

#### 2.2.2. Rat Sarcoma Virus (Ras)/Rapidly Accelerated Fibrosarcoma (Raf)/Mitogen-Activated Protein Kinase Kinase (MEK)/Extracellular Signal-Regulated Kinase (ERK) Pathway

The Ras/Raf/MEK/ERK pathway is a key downstream cascade of receptor tyrosine kinases (RTKs), such as VEGFRs and FGF receptors (FGFRs) [141,142]. The binding of ligands to RTKs stimulates the activation of Ras, which is a small GTPase [143]. Activated Ras then recruits and phosphorylates the serine/threonine kinase Raf, which promotes MEK activation and ERK phosphorylation. Phosphorylated ERK translocates into the cell nucleus, where it regulates the activity of various transcription factors and the expression of different genes [143,144]. Liu et al. found that miR-7-5p (miR-7) directly targets *RAF1*, thereby inhibiting HUVEC proliferation [91]. Furthermore, they observed a reduction in miR-7 expression and a negative correlation between the expression of this miRNA and RAF1 in the microvasculature of human glioblastoma tissues [91]. Recent studies have also reported the targeting of *RAF1* by miR-7 in lymphoma cells [145], lung epithelial cells [146] and breast cancer cells [147]. The miRNA-302-367 cluster, composed of miR-302a-3p (miR-302a), miR-302b-3p (miR-302b), miR-302c-3p (miR-302c), miR-302d-3p (miR-302d) and miR-367-3p (miR-367), has been reported to suppress HUVEC sprouting and migration by targeting *ERK1* and *ERK2* [77]. The expression of this miRNA cluster was decreased in HUVECs that were cocultured with LLC1 Lewis lung carcinoma. However, the endothelial-specific overexpression of miRNA-302-367, achieved by generating miR-302-367^ECTg^ mice or using Arg-Gly-Asp (RGD) peptide-containing magnetic nanoparticles to deliver miR-302-367 mimics to ECs reduced tumor growth by restricting angiogenesis, offering a novel strategy for anti-cancer therapy [77].

#### 2.2.3. Phosphoinositide 3-Kinase (PI3K)/Protein Kinase B (AKT) Pathway

Another downstream cascade of RTKs that plays an essential role in angiogenesis is the PI3K/AKT pathway [148]. Upon growth factor stimulation, activated receptors recruit and phosphorylate PI3K [149]. This leads to the activation of PI3K, which, in turn, catalyzes the conversion of phosphatidylinositol (3,4)-bisphosphate (PIP2) to phosphatidylinositol (3,4,5)-trisphosphate (PIP3) [149]. PIP3 then binds to AKT and recruits it to the plasma membrane, where AKT is sequentially phosphorylated by 3-phosphoinositide-dependent protein kinase 1 (PDK1) and PDK2 [148]. Activated AKT phosphorylates its downstream angiogenesis-related substrates, such as mechanistic target of Rapamycin (mTOR) and endothelial nitric oxide synthase (eNOS) [150]. 

Phosphatase and tensin homolog (PTEN), a phosphatase that converts PIP3 to PIP2, inhibits AKT activation and acts as a negative regulator of PI3K/AKT signaling. Its mRNA can be targeted by multiple endothelial miRNAs, including miR-130b-3p, miR-181b-5p (miR-181b), miR-205-5p (miR-205), miR-23a-3p (miR-23a), miR-26a-5p (miR-26a) and miR-494-3p (miR-494). As a consequence, these miRNAs activate AKT activation in ECs and stimulate angiogenesis in different types of cancer [31,46,51,67,70,84]. Among these miRNAs, miR-205 has been well studied in vitro and vivo. According to He et al. [51], miR-205 was enriched in TECs and correlated positively with high microvessel density in ovarian cancer patients. Exosomal miR-205 from ovarian cancer cells promoted HUVEC angiogenesis by regulating the PTEN-AKT pathway and accelerated tumor angiogenesis and growth in a mouse xenograft tumor model [51]. The suppression of PTEN by miR-205 was consistent with a previous study, which confirmed PTEN as a direct target of miR-205 using luciferase reporter assays [151].

Sirtuin 1 (SIRT1) also plays an important role in regulating AKT activation [152]. It is a nicotinamide adenine dinucleotide-dependent class III histone deacetylase, which deacetylates AKT and promotes its binding to PIP3 [152]. Endothelial miR-22 and miR-23a have been reported to target *SIRT1*, leading to the inhibition of angiogenesis [59,66]. In fact, the direct binding of miR-22 to 3′UTR of *SIRT1* has been intensively demonstrated in many studies [153,154,155,156].

#### 2.2.4. Krüppel-Like Factors (KLFs)

KLFs are a family of zinc finger-containing transcription factors that regulate basic cellular processes, including apoptosis, proliferation, migration, differentiation, inflammation and metabolism [157]. They are involved in the pathophysiology of diverse diseases, such as obesity and cancer [157]. So far, researchers have identified 18 different KLFs, among which KLF2, KLF4, KLF5 and KLF10 play important roles in regulating angiogenesis [158,159,160,161]. Previous studies have shown that exosomal miR-182-5p (miR-182) secreted by hypoxic glioblastoma cells and exosomal miR-25 derived from colorectal cancer cells induce angiogenesis and increase vascular permeability by targeting *KLF2* and *KLF4* in ECs [47,69]. The direct binding of miR-25 to *KLF4* was further confirmed by Lu et al. [162] using a dual-luciferase reporter assay. Additionally, *KLF2* is also a bona fide target of pro-angiogenic miR-3157-3p and miR-92a [78,97]. Ling and colleagues first proved that miR-92a targets *KLF2* [163]. In addition, a recent study has reported that miR-141 secreted by small-cell lung cancer cells is able to be delivered to ECs via exosomes and promote EC angiogenic activity by targeting *KLF12* [35]. However, the specific role of KLF12 in tumor angiogenesis needs further elucidation.

#### 2.2.5. TGF-β/TGF-β Receptor (TGFBR) Pathway

TGF-β signals by assembling a hetero-tetrameric receptor complex composed of two TGFBR1s (also called ALK5) and two TGFBR2s [164]. TGFBR2 phosphorylates and activates TGFBR1, which subsequently activates receptor-associated SMADs (RSMADs), i.e., SMAD2 and SMAD3. These RSMADs form a complex with SMAD4 and translocate into the nucleus, where they activate or repress the transcription of target genes [116,165]. As mentioned above, TGF-β signaling plays a pivotal role in tumor angiogenesis [166], making it an ideal target for angiogenesis-associated miRNAs. Endothelial miR-142-3p and miR-210-3p have been shown to directly inhibit the expression of *TGFBR1* and *SMAD4*, respectively, resulting in enhanced angiogenesis [37,54]. The relationship between miR-142-3p and *TGFBR1* has been well established in several other cell types, including NSCLC cells, M2 macrophages, oral cancer cells and hepatic stellate cells [167,168,169,170]. Additionally, it has also been confirmed that miR-210-3p binds to the 3′UTR of *SMAD4* [171,172].

#### 2.2.6. Suppressor of Cytokine Signaling (SOCS)

SOCS proteins are negative regulators of the Janus kinase (JAK)/signal transducer and activator of transcription (STAT) pathway [173]. The evolutionarily conserved JAK/STAT pathway regulates a variety of developmental and homeostatic processes, such as the development of the immune system, hematopoiesis and stem cell maintenance [174]. Growing evidence suggests that this pathway significantly contributes to tumor angiogenesis by promoting EC survival, proliferation and migration [175,176]. The JAK/STAT pathway is initiated upon the binding of cytokines or growth factors to their specific receptor subunits. This leads to the multimerization of the receptor subunits and the transphosphorylation of receptor-associated JAKs. Activated JAKs, in turn, phosphorylate the cytoplasmic tyrosine residues of receptors to provide docking sites for STATs. Phosphorylated STATs dimerize and translocate to the nucleus, where they regulate the transcription of diverse genes [174]. SOCS is capable of downregulating the JAK/STAT pathway via different mechanisms. These include blocking the binding of STAT to receptor, directly inhibiting the kinase activity of JAK and promoting the degradation of JAK or STAT [173]. Accordingly, SOCS acts as an angiogenesis inhibitor. The targeting of *SOCS3* and *SOCS5* by endothelial miR-221, miR-141-3p (miR-141) and miR-9, respectively, activates the JAK/STAT pathway and consequently stimulates the formation of new blood vessels [36,62,93]. Additional studies further confirm the binding relationship between miR-221 and the 3′UTR of SOCS3 [177,178], as well as between miR-9 and the 3′UTR of SOCS5 [179,180].

#### 2.2.7. Matrix Metalloproteinases (MMPs)

MMPs, a family of zinc-dependent endopeptidases, facilitate tumor angiogenesis and metastasis by degrading components of the extracellular matrix (ECM), resulting in the release of ECM-sequestered pro-angiogenic factors and exposure of the integrin-binding sites of ECM proteins [181,182]. On the contrary, tissue inhibitors of MMPs (TIMPs) are known to inhibit the activity of MMPs and act as negative regulators of angiogenesis [183]. Liu et al. recently reported that miR-526b-3p targets *MMP2* and *VEGF* in ECs cultured in glioma cell-conditioned medium (GECs), causing a significant decrease in GEC viability, migration and tube formation [87]. In addition, the downregulation of TIMP2 by miR-3157-3p in ECs contributed to its pro-angiogenic effects in NSCLC [78].

## 3. Therapeutic Applications of Endothelial miRNAs

So far, several anti-angiogenic agents have been approved by the United States Food and Drug Administration (FDA) for the treatment of metastatic cancers, such as colorectal cancer, renal cell carcinoma, hepatocellular carcinoma and thyroid cancer. These agents include humanized monoclonal antibodies against VEGF/VEGFR (e.g., bevacizumab and ramucirumab), the soluble VEGF decoy receptor aflibercept as well as tyrosine kinase inhibitors (e.g., sunitinib and sorafenib) [184]. Unfortunately, their clinical efficiency is quite low due to the onset of innate or acquired resistance [184,185]. This resistance is mediated by different mechanisms, including the elevation of intratumoral hypoxia, the upregulation of alternative angiogenic pathways and increased tumor metastasis [184]. Therefore, it is necessary to search for more effective strategies for anti-angiogenic cancer therapy.

Given their potent regulatory function in TEC activity, endothelial miRNAs represent promising novel targets for the development of second-generation anti-angiogenic therapeutics. In this context, two major approaches have been suggested in endothelial miRNA-based therapy [186,187]. There is the possibility of introducing anti-angiogenic miRNAs into TECs. On the other hand, TECs can be treated with miRNA antagonists (also called antagomirs or anti-miRNAs) that inhibit pro-angiogenic miRNAs. However, the cellular uptake of miRNAs or antagonists is hampered by their charge repulsion and high vulnerability to serum RNase degradation. To overcome this problem, chemical modifications and sophisticated delivery systems have been established in recent years [188].

Chemical modifications of miRNAs or anti-miRNAs include phosphorothioate backbone modification, 2′-O-methyl conjugation or locked nucleic acid (LNA) modification [189]. Unfortunately, these chemical structure optimizations only slightly improve the stability and cellular penetration of RNA oligonucleotides. In contrast, non-viral (i.e., lipids, polymers, inorganic compounds and extracellular vesicles) and viral delivery systems (i.e., lentivirus and adeno-associated virus (AAV)) successfully protect oligonucleotides from nuclease degradation and transport them to different organs, such as the liver and the kidneys [189,190]. Based on these delivery systems, considerable progress has been made in the selective transport of miRNAs or anti-miRNAs to ECs, which are particularly difficult to transfect or transduce.

EC-targeting peptides conjugated to lipid- and polymer-based nanoparticles are most widely used. For instance, the systemic administration of anti-miR-132-3p (miR-132), anti-miR-296 and miR-7 loaded in nanoparticles modified with cyclic RGD has been shown to increase the endothelial uptake of oligonucleotides and inhibit the angiogenic activity of ECs in vitro and in vivo [191,192,193]. Of note, RGD is a peptide that binds to integrin αvβ3 and αvβ5 on the membrane of ECs. More recently, RGD-modified exosomes overexpressing miR-92b-3p (miR-92b) were found to inhibit ovarian cancer angiogenesis and growth [98]. Similarly, the Ala-Pro-Arg-Pro-Gly (APRPG) peptide, which has an affinity to VEGFR1 on ECs, was utilized to generate APRPG-polyethylene glycol (PEG)-modified lipoplexes for the in vivo delivery of miR-499-5p (miR-499) to tumors via intravenous injection. These miRNA-carrying lipoplexes accumulated in tumor blood vessels and inhibited the growth of colon carcinoma [194]. Moreover, the integrin α4β1 ligand Arg-Glu-Asp-Val (REDV) was linked to trimethyl chitosan via a PEG linker. This modified polyplex selectively delivered miR-126 to ECs and consequently enhanced their proliferation [195].

The screening of random peptide libraries on the surface of AAV capsids has been performed to identify vectors that enable high transduction efficiency in ECs. One successful example of such a vector is the modified AAV9 capsid plasmid displaying peptide SLRSPPS [196]. By using this modified vector, the overexpression of miR-92a significantly inhibited endothelium-dependent relaxation in mouse aortas [197].

Nonetheless, despite the above-mentioned achievements, the efficient, specific and safe delivery of miRNAs or anti-miRNAs to ECs, especially TECs, still remains a big challenge to date.

## 4. Concluding Remarks and Perspectives

Over the last two decades, there has been significant interest in the role of miRNAs in tumor angiogenesis, leading to intensive research in this field. Our comprehensive search of the literature on PubMed revealed that approximately 80% of publications focus on the indirect effects of miRNAs in tumor cells on EC angiogenesis. However, ECs are the primary cell type responsible for angiogenesis. Therefore, our systematic review specifically focused on endothelial miRNAs that play crucial roles in regulating the aberrant angiogenic activity of TECs. The definitions of TECs in publications can be categorized into different groups: (i) ECs cultured with tumor cell-conditioned medium; (ii) ECs co-cultured with tumor cells without direct contact on a Transwell plate; (iii) ECs co-cultured with tumor cells with direct contact and subsequently isolated from tumor cells; (iv) ECs isolated from fresh human or mouse tumor tissues; and (v) ECs isolated from formalin-fixed paraffin-embedded (FFPE) human tumor samples using laser capture microdissection. Although all the TEC types mentioned above have been considered in this review, it is important to note that the in vitro experimental settings used to study TECs only monitor a fraction of the TME. The TME, characterized by hypoxia, acidity and nutrient deficiency, contains not only tumor cells but also immune cells, fibroblasts, macrophages and the extracellular matrix [6]. In our view, ECs dissected from FFPE human tumor tissues best capture the features of TECs in the TME. Even freshly isolated TECs from tumor tissues, while still valuable for analysis, are no longer considered true TECs as they have been removed from the TME.

Previous studies have shown that the intracellular levels of endothelial miRNAs involved in tumor angiogenesis are mainly determined by the TME via hypoxia, pro-angiogenic factors, cell–cell transfer and sponging by lncRNAs and circRNAs. Moreover, these miRNAs target key angiogenesis-related signaling pathways or proteins, including the VEGF/VEGFR, Ras/Raf/MEK/ERK, PI3K/AKT and TGF-β/TGFBR pathway, as well as KLFs, SOCS and MMPs/TIMPs. While we present these mechanisms of endothelial miRNA regulation and function separately in this review for organizational purposes, it should be noted that they interconnect with each other and form a complex network. For instance, studies have shown that hypoxia can stimulate the expression of pro-angiogenic factors such as VEGF, IL-1β and TGF-β in a variety of cell types [198,199,200,201,202,203]. Therefore, it is not surprising that miR-1, miR-22 and miR-30c, which have been reported to be downregulated by VEGF, IL-1β and TGF-β in ECs, respectively [21,59,76], could also be inhibited by hypoxia in certain scenarios [204,205,206,207,208]. Moreover, KLF2 has been shown to suppress the expression of VEGFR2 by inhibiting its promoter activity [158]. Accordingly, KLF2-targeting miR-182 and miR-25 upregulate VEGFR2 in ECs and consequently promote angiogenesis [47,69].

This systematic review highlights the critical roles of endothelial miRNAs in regulating tumor angiogenesis. Furthermore, the multiple-gene-targeting capacity of miRNAs may help to prevent acquired therapy resistance. Therefore, targeting endothelial miRNAs holds promise as a novel approach for developing second-generation anti-angiogenic cancer treatments. Combining endothelial miRNA-targeting therapy with other anti-cancer treatments, such as chemotherapy, radiotherapy and immunotherapy, may enhance clinical outcomes. However, due to the nascent stage of therapeutic applications for endothelial miRNAs, several scientific and technical challenges must be addressed. To facilitate clinical translation, a better understanding of the regulatory and functional networks of endothelial miRNAs is a critical prerequisite. Moreover, the development of efficient and safe miRNA delivery systems specific to TECs is required. In addition, it is essential to assess the effects, dosage, pharmacokinetics, side effects and acquired resistance of endothelial miRNA-targeting treatments in appropriate animal models. Rapid and significant progress in RNA sequencing technologies enabling the discovery of new miRNAs, high-throughput approaches for miRNA target identification, chemical modifications of miRNAs, nanotechnology and viral vector development, as well as tailored animal models for drug discovery and development, may help researchers achieve these goals in the near future.

## Figures and Tables

**Table 1:** Regulation and function of endothelial miRNAs in tumor angiogenesis (the previous miRNA names are given in brackets when available in miRBase).

Endothelial miRNAs	Regulation	Function			Tumor Type	Ref
		Target	Downstream Pathway	Pro- or Anti-Angiogenic Action		
miR-1-3p (miR-1)	Downregulated by VEGF	*MPL*	Inhibition of ERK1 and 2 phosphorylation	anti	NSCLC	[21]
miR-10a-3p	Transferred via tumor cell (TC)-derived exosomes	*ZMYND11*	n.a.	pro	gastric cancer	[22]
miR-103a-3p (miR-103a)	Sponged by circ-DICER1	*ZIC4*	Downregulation of Hsp90β	anti	glioma	[23]
miR-1229-3p (miR-1229)	Transferred via TC-derived exosomes	*HIPK2*	Activation of VEGF pathway	pro	colorectal cancer	[24]
miR-124-3p (miR-124)	Sponged by TC-derived exosomal circHIPK3	*MTDH*	n.a.	anti	breast cancer	[25]
miR-1246	Transferred via TC-derived microvesicles	*PML*	Activation of Smad1, 5 and 8 signaling	pro	colorectal cancer	[26]
miR-125b-5p (miR-125b)	Downregulated by VEGF	*MAZ*	Downregulation of VEGF	anti	glioblastoma	[27]
miR-126-3p (miR-126)	Downregulated by co-culture with cervical cancer cells and fibroblasts	*ADM*	n.a.	anti	cervical cancer	[28]
miR-1260b	Transferred via TC-derived exosomes	*HIPK2*	n.a.	pro	NSCLC	[29]
miR-1290	Transferred via TC-derived exosomes	*SMEK1*	Upregulation of VEGFR2 phosphorylation	pro	hepatocellular carcinoma (HCC)	[30]
miR-130b-3p	Transferred via TC-derived exosomes	*PTEN*	n.a.	pro	oral squamous cell carcinoma (OSCC)	[31]
miR-135b-5p (miR-135b)	Transferred via hypoxic TC-derived exosomes	*FIH-1*	Upregulation of HIF1 transcriptional activity	pro	multiple myeloma	[32]
miR-138-5p (miR-138)	Sponged by circ_002136	*SOX13*	Upregulation of SPON2	anti	glioma	[33]
miR-141-3p (miR-141)	Transferred via TC-derived exosomes	*GAX*	n.a.	pro	lung cancer	[34]
	Transferred via TC-derived exosomes	*KLF12*	n.a.	pro	small-cell lung cancer	[35]
	Transferred via TC-derived exosomes	*SOCS5*	Activation of JAK/STAT3 and NF-κB pathways; upregulation of VEGFR2	pro	ovarian cancer	[36]
miR-142-3p	Transferred via TC-derived extracellular vesicles	*TGFBR1*	n.a.	pro	NSCLC	[37]
miR-143-3p (miR-143)	Transferred via TC-derived exosomes	*CAMK1D*	n.a.	pro	lung cancer	[38]
miR-144-3p (miR-144)	Transferred via TC-derived extracellular vesicles	*FBXW7*	Upregulation of HIF1α/VEGF signaling	pro	nasopharyngeal carcinoma (NPC)	[39]
miR-145-5p (miR-145)	Transferred via TC-derived exosomes	*CAMK1D*	n.a.	pro	lung cancer	[38]
miR-146a-5p (miR-146a)	Upregulated via indirect co-culture with TCs	*BRCA1*	Upregulation of PDGFRA expression	pro	HCC	[40]
miR-148a-3p (miR-148a)	Transferred via TC-derived exosomes	n.a.	Upregulation of VEGF, IL-6 and IL-8	pro	osteosarcoma	[41]
	Transferred via TC-derived exosomes	*ERRFI1*	Activation of EGFR/ERK pathway	pro	glioma	[42]
miR-153-3p	Sponged by TC-derived exosomal lncRNA SNHG15	*VEGF*; *CDC42*	n.a.	anti	glioma	[43]
miR-17-5p (miR-17)	Transferred via TC-derived exosomes	*BAMBI*	Upregulation of AKT phosphorylation and VEGF expression	pro	NPC	[44]
miR-181a-5p (miR-181a)	Transferred via hypoxic TC-derived exosomes	*MLL3*	Upregulation of YAP/VEGF pathway	pro	papillary thyroid cancer	[45]
miR-181b-5p (miR-181b)	Transferred via TC-derived extracellular vesicles	*PTEN*; *PHLPP2*	Activation of AKT signaling	pro	esophageal squamous cell carcinoma	[46]
miR-182-5p (miR-182)	Transferred via hypoxic TC-derived exosomes	*KLF2*; *KLF4*	Accumulation of VEGFR2	pro	glioblastoma	[47]
	Transferred via TC-derived extracellular vesicles	*CMTM7*	Activation of EGFR/AKT signaling	pro	breast cancer	[48]
miR-186-5p (mir-186)	Downregulated by hypoxia	*PRKCA*	Upregulation of ERK phosphorylation	anti	NSCLC	[49]
	Sponged by lncRNA PVT1	*ATG7*; *BECN1*	n.a.	anti	glioma	[50]
miR-205-5p (miR-205)	Transferred via TC-derived exosomes	*PTEN*	Activation of AKT	pro	ovarian cancer	[51]
miR-21-5p (miR-21)	Transferred via cancer stem cell-derived exosomes	n.a.	Activation of VEGF/VEGFR2 pathway	pro	glioblastoma	[52]
	Transferred via TC-derived exosomes	*KRIT1*	Activation of β-catenin pathway and upregulation of VEGF and Ccnd1	pro	colorectal cancer	[53]
	Transferred via TC-derived exosomes	n.a.	Upregulation of VEGF, IL-6 and IL-8	pro	osteosarcoma	[41]
miR-210-3p	Transferred via TC-derived exosomes	*SMAD4*; *STAT6*	n.a.	pro	HCC	[54]
	Transferred via TC-derived exosomes	*EFNA3*	n.a.	pro	breast cancer	[55]
	Transferred via hypoxic TC-derived exosomes	*EFNA3*	n.a.	pro	leukemia	[56]
	Transferred via TC-derived exosomes	*EFNA3*	Activation of PI3K/AKT pathway	pro	OSCC	[57]
miR-218-5p (miR-218)	Downregulated in TECs	*ROBO1*	n.a.	anti	gastric cancer	[58]
miR-22-3p (miR-22)	Downregulated by IL-1β	*SIRT1*; *FGFR1*	Inactivation of AKT/mTOR signaling	anti	NSCLC	[59]
miR-221-3p (miR-221)	Transferred via TC-derived exosomes	*PIK3R1*	n.a.	pro	OSCC	[60]
	Transferred via TC-derived exosomes	*THBS2*	n.a.	pro	cervical squamous cell carcinoma	[61]
	Transferred via TC-derived extracellular vesicles	*SOCS3*	Upregulation of STAT3/VEGFR2 signaling	pro	colorectal cancer	[62]
	Transferred via TC-derived exosomes	*MAPK10*	Downregulation of c-FOS, c-JUN and JUNB; upregulation of VEGF	pro	cervical cancer	[63]
miR-23a-3p (miR-23a)	Transferred via TC-derived exosomes	*TSGA10*	n.a.	pro	NPC	[64]
	Transferred via hypoxic TC-derived exosomes	*PHD1*; *PHD2*; *ZO-1*	Accumulation of HIF1α	pro	lung cancer	[65]
	Transferred via hypoxic TC-derived exosomes	*SIRT1*	n.a.	pro	HCC	[66]
	Transferred via TC-derived extracellular vesicles	*PTEN*	Upregulation of AKT and ERK phosphorylation	pro	lung cancer	[67]
miR-2355-5p (miR-2355)	Sponged by TC-derived exosomal lncRNA RAMP2-AS1	*VEGFR2*	n.a.	anti	chondrosarcoma	[68]
miR-25-3p (miR-25)	Transferred via TC-derived exosomes	*KLF2*; *KLF4*	Upregulation of VEGFR2	pro	colorectal cancer	[69]
miR-26a-5p (miR-26a)	Transferred via cancer stem cell-derived exosomes	*PTEN*	Activation of PI3K/AKT pathway	pro	glioma	[70]
miR-27a-3p (miR-27a)	Transferred via TC-derived exosomes	*BTG2*	Upregulation of VEGF, VEGFR, MMP2 and MMP9	pro	pancreatic cancer	[71]
	Transferred via TC-derived exosomes	*SFRP1*	Upregulation of VEGF and TNFα	pro	renal clear cell carcinoma	[72]
miR-29a-3p (miR-29a)	Sponged by lncRNA H19	*VASH2*	n.a.	pro	glioma	[73]
miR-296-5p (miR-296)	Upregulated by VEGF	*HGS*	Upregulation of VEGFR2 and PDGFRβ	pro	glioma	[74]
miR-30b-5p (miR-30b)	Transferred via hypoxic TC-derived exosomes	*GJA1*	n.a.	pro	pancreatic cancer	[75]
miR-30c-5p (miR-30c)	Downregulated by TGF-β	*SERPINE1*	n.a.	anti	breast cancer	[76]
miRNA-302-367 cluster	Downregulated via indirect co-culture with TCs	*ERK1*; *ERK2*	Upregulation of KLF2, S1pr1 and VE-cadherin expression	anti	lung cancer	[77]
miR-3157-3p	Transferred via TC-derived exosomes	*TIMP2*; *KLF2*	Upregulation of VEGF, MMP2 and MMP9	pro	NSCLC	[78]
miR-3178	Downregulated in TECs	*EGR3*	n.a.	anti	HCC	[79]
miR-3619-5p (miR-3619)	Sponged by TC-derived exosomal circCMTM3	*SOX9*	n.a.	anti	HCC	[80]
miR-382-5p (miR-382)	Sponged by circ-DICER1	*ZIC4*	Downregulation of Hsp90β	anti	glioma	[23]
miR-383-5p	Downregulated in TECs	*VEGF*	Inhibition of FAK and Src pathways	anti	glioma	[81]
miR-4488	Transferred via TC-derived exosomes	*CX3CL1*	n.a.	anti	breast cancer	[82]
miR-4500	Sponged by TC-derived exosomal lnRNA SNHG16	*GALNT1*	Inhibition of PI3K/AKT/mTOR pathway	anti	HCC	[83]
miR-494-3p (miR-494)	Transferred via TC-derived microvesicles	*PTEN*	Activation of AKT/eNOS pathway	pro	NSCLC	[84]
	Transferred via TC-derived exosomes	*PTPN12*	Phosphorylation of ERK and eNOS	pro	lung cancer	[85]
miR-5096	Transferred through TC gap junction	n.a.	Upregulation of connexin 43	pro	glioblastoma	[86]
miR-526b-3p	Sponged by circ-ATXN1	*MMP2*; *VEGF*	n.a.	anti	glioma	[87]
miR-549a	Transferred via TC-derived exosomes	*HIF1A*	n.a.	anti	renal cancer	[88]
miR-584-5p (miR-584)	Transferred via TC-derived extracellular vesicles	*PCK1*	Activation of NRF2	pro	HCC	[89]
miR-663b	Transferred via TC-derived exosomes	*VCL*	n.a.	pro	cervical cancer	[90]
miR-7-5p (miR-7)	Downregulated in glioblastoma microvasculature	*RAF1*	n.a.	anti	glioblastoma	[91]
miR-9-5p (miR-9)	Transferred via TC-derived exosomes	n.a.	n.a.	pro	glioma	[92]
	Transferred via TC-derived microvesicles	*SOCS5*	Activation of JAK/STAT pathway	pro	NSCLC; melanoma; pancreatic cancer; glioblastoma; colorectal cancer	[93]
	Transferred via epithelial cell-derived exosomes	*MDK*	Inhibition of PDK/AKT signaling	anti	NPC	[94]
miR-92a-3p (miR-92a)	Transferred via TC-derived exosomes	*DKK3*	n.a.	pro	colorectal cancer	[95]
	Transferred via TC-derived exosomes	*ITGA5*	n.a.	pro	leukemia	[96]
	Transferred via TC-derived exosomes	*KLF2*	Upregulation of IL-1, IL-6, IL-8, MCP-1, VCAM1 and ICAM1	pro	retinoblastoma	[97]
miR-92b-3p (miR-92b)	Transferred via ovarian epithelial cell-derived exosomes	*SOX4*	Downregulation of endothelin-1 expression and AKT phosphorylation	anti	ovarian cancer	[98]
miR-940	Transferred via TCs-derived exosomes	*ETS1*	Downregulation of VEGFR2	anti	HCC	[99]
miR-944	Transferred via cancer stem cell-derived exosomes	*VEGF-C*	Inhibition of AKT and ERK pathways	anti	glioma	[100]
miR-96-5p (miR-96)	Sponged by hypoxic TC-derived exosomal lncRNA UCA1	*AMOLT2*	Downregulation of ERK phosphorylation	anti	pancreatic cancer	[101]

n.a.: not available.

## Data Availability

Data sharing not applicable.
